# International Radiotherapy Referrals From Rural Rwanda: Implementation Processes and Early Clinical Outcomes

**DOI:** 10.1200/JGO.18.00089

**Published:** 2018-10-15

**Authors:** Maud Hanappe, Lowell T. Nicholson, Shekinah N.C. Elmore, Alexandra E. Fehr, Jean Bosco Bigirimana, Ryan C. Borg, John Butonzi, Cyprien Shyirambere, Egide Mpanumusingo, Marie O. Benewe, Dan M. Kanyike, Scott Triedman, Lawrence N. Shulman, Christian Rusangwa, Paul H. Park

**Affiliations:** **Maud Hanappe**, Free University of Brussels, Brussels, Belgium; **Lowell T. Nicholson**, Duke University School of Medicine, Durham, NC; **Shekinah N.C. Elmore**, Harvard Radiation Oncology Program; **Shekinah N.C. Elmore** and **Paul H. Park**, Harvard Medical School; **Ryan C. Borg**, Boston College School of Social Work; **Paul H. Park**, Brigham and Women’s Hospital; **Paul H. Park**, Partners in Health, Boston, MA; **Alexandra E. Fehr**, **Jean Bosco Bigirimana**, **Cyprien Shyirambere**, **Egide Mpanumusingo**, **Marie O. Benewe**, and **Christian Rusangwa**, Inshuti mu Buzima/Partners in Health; **John Butonzi**, Butaro District Hospital, Rwanda Ministry of Health, Butaro, Rwanda; **Dan M. Kanyike**, Uganda Cancer Institute, Kampala, Uganda; **Scott Triedman**, The Warren Alpert Medical School of Brown University, Providence, RI; and **Lawrence N. Shulman**, University of Pennsylvania, Philadelphia, PA.

## Abstract

**Purpose:**

Low- and middle-income countries disproportionately comprise 65% of cancer deaths. Cancer care delivery in resource-limited settings, especially low-income countries in sub-Saharan Africa, is exceedingly complex, requiring multiple modalities of diagnosis and treatment. Given the vast human, technical, and financial resources required, access to radiotherapy remains limited in sub-Saharan Africa. Through 2017, Rwanda has not had in-country radiotherapy services. The aim of this study was to describe the implementation and early outcomes of the radiotherapy referral program at the Butaro Cancer Centre of Excellence and to identify both successful pathways and barriers to care.

**Methods:**

Butaro District Hospital is located in a rural area of the Northern Province and is home to the Butaro Cancer Centre of Excellence. We performed a retrospective study from routinely collected data of all patients with a diagnosis of cervical, head and neck, or rectal cancer between July 2012 and June 2015.

**Results:**

Between 2012 and 2015, 580 patients were identified with these diagnoses and were potential candidates for radiation. Two hundred eight (36%) were referred for radiotherapy treatment in Uganda. Of those referred, 160 (77%) had cervical cancer, 31 (15%) had head and neck cancer, and 17 (8%) had rectal cancer. At the time of data collection, 101 radiotherapy patients (49%) were alive and had completed treatment with no evidence of recurrence, 11 (5%) were alive and continuing treatment, and 12 (6%) were alive and had completed treatment with evidence of recurrence.

**Conclusion:**

This study demonstrates the feasibility of a rural cancer facility to successfully conduct out-of-country radiotherapy referrals with promising early outcomes. The results of this study also highlight the many challenges and lessons learned in providing comprehensive cancer care in resource-limited settings.

## INTRODUCTION

The burden of cancer continues to have a global impact across low- and high-income countries and among both urban and rural populations. Furthermore, cancer is the leading cause of premature death worldwide.^1^ However, the distribution of mortality is not uniform across all demographics. Low- and middle-income countries (LMICs) disproportionately comprise 65% of cancer deaths, driven in large part by rates within sub-Saharan Africa (SSA), where cancer mortality is expected to double by 2030.^2^

A myriad of factors contribute to the disproportionately high cancer mortality rate in SSA. Late stage at presentation and limited access to treatment heighten the risk of mortality.^3^ More broadly, there is also a fundamental clinical reality that cancer treatment in any part of the world is complex and the limited resources available in SSA further accentuate this challenge. Radiotherapy itself, perhaps more so than other essential components of cancer care delivery, has distinct implementation challenges related to the cost of machine purchase; the technical expertise in radiation oncologists, therapists, physicists, and radiation nurses; and maintenance. Because radiotherapy is often viewed as too expensive and too complex, it is frequently omitted from cancer centers in LMICs.^4,5^ Consequently, access to high-quality radiotherapy in LMICs remains extremely limited. It is estimated that only 28% of current radiotherapy needs are being met in Africa, with the majority (60%) of the available radiotherapy capacity being located in Egypt and South Africa.^6,7^

Rwanda is one of the 28 African countries that currently does not have a radiotherapy facility.^8^ Access to radiotherapy for Rwandans has been limited historically to those who are able to travel abroad, either to neighboring countries with radiotherapy capacity (eg, Kenya, and Tanzania), or further afield to India or Europe..^9^ The cost of such services has limited access to those with substantial wealth and to the few patients sponsored by the Rwandan National Referral Board each year.

In July 2012, the Rwandan Ministry of Health (MOH), with support from Partners In Health (PIH), Dana-Farber/Brigham and Women’s Cancer Center (DFBWCC), and other key partners, opened a national cancer referral center at Butaro District Hospital, located in the rural Northern Province.^8,10^ The Butaro Cancer Centre of Excellence (BCCOE) brings comprehensive cancer care, including clinical and pathology services and diagnosis, chemotherapy, surgery, patient follow-up, palliative care, and mental health and socioeconomic support, to rural East Africa. Given economic constraints, BCCOE currently does not offer radiotherapy services. As a result, BCCOE also began providing financial support to a limited number of patients requiring potentially curative radiotherapy and referred these patients to the Uganda Cancer Institute (UCI) in Kampala, Uganda, for radiotherapy.^8,10,11^

Despite a documented need for expanded access to radiotherapy in LMICs, limited information exists regarding radiotherapy implementation processes, treatment protocols, or outcomes in resource-limited settings.^4^ This lack of evidence is especially true for patients who, like most in SSA, do not have locally available radiotherapy services. Here we describe a novel implementation approach to providing life-saving radiotherapy services to patients with cancer in rural Rwanda. The study describes the baseline characteristics, preliminary clinical outcomes, and lessons learned from the BCCOE experience of international radiotherapy referrals.

## METHODS

### Setting and Implementation Design

The BCCOE is located 90 km north of the capital city Kigali in Burera District and is adjacent to the country’s northern border with Uganda. The majority of patients live in rural and mostly agriculture-based settings that are outside the district catchment area. As described elsewhere, BCCOE provides comprehensive care, from initial pathology to curative treatment and palliative care, with the exception of radiotherapy.^8,10,11^

All diagnosis, staging, and treatment protocols at BCCOE follow guidelines set forth by the Rwandan MOH. The creation of the clinical protocols was supported by oncologists at DFBWCC. Unique to the resource-limited setting, the initial cervical cancer protocol through 2013 did not require pathologic confirmation, as was standard of care across multiple SSA countries.^12^ In addition, rectal cancer was staged using a rectal examination, a colonoscopy, a computed tomography scan of the abdomen and pelvis, and an x-ray of the chest and then classified as early stage (surgeon feels that the rectal cancer is easily resectable, no distant metastases), locally advanced (rectal tumor is not easily resectable, no distant metastases), or metastatic. Similar staging categories were present for head and neck cancer, whereby a physical examination and computed tomography scan provided data for staging criteria. Regarding radiotherapy treatment, clinical guidelines were based on that of the UCI. The radiotherapy technology available included both a cobalt external-beam therapy unit and intracavitary brachytherapy capacity.

Clinical and programmatic staff at BCCOE produced a standard operating procedure for radiotherapy candidate evaluation, selection, transfer, and follow-up. On a monthly basis, 10 to 15 patients were selected to receive fully subsidized radiotherapy on the basis of predefined criteria, which included curability, age, and Eastern Cooperative Oncology Group (ECOG) performance status. All patients required an ECOG performance status ≤ 2. This requirement was set both for prognostic purposes and because patients treated at UCI had to be ambulatory and capable of self-care while undergoing their outpatient treatment regimens. After selection for radiotherapy referral, patients were required to complete a repeat clinical evaluation to confirm clinical stability and stage, given concerns about possible disease progression between initial diagnosis and initiation of radiotherapy. On conclusion of the clinical evaluation, patients would then be instructed on travel details and requirements, including the need to obtain travel documents to enter Uganda. Patients would also receive a follow-up clinic appointment for ongoing management after the completion of radiotherapy. An oncology nurse would accompany the patient group by bus from Kigali to UCI for the 9-hour trip. On arrival, the nurse oriented the patients to the living accommodations at a hostel, as well as to the clinical facilities at UCI. In addition, the nurse delivered Rwandan MOH clinical referral documents to the UCI staff. Each patient would then receive an individualized initial evaluation and treatment plan. The initial evaluation sometimes included additional radiographic studies surrounding disease stage. In the case of medical emergencies, patients would be admitted promptly as needed. On completion of radiation treatment, a discharge summary report would be prepared for each patient, and the patient group would be accompanied back to Kigali by a Rwandan nurse.

### Study Design

This is a retrospective study of all patients at BCCOE with a diagnosis of cervical, head and neck, or rectal cancer between July 1, 2012, and June 30, 2015. These three cancers were chosen for analysis because they were the most common cancers referred for radiotherapy as determined by internal monitoring and evaluation reports at BCCOE. Patients who met these inclusion criteria were identified using the electronic medical record system, (OpenMRS, Indianapolis, IN). Patients were excluded if they had missing paper records or records without a documented clinical or pathologic diagnosis. Supplemental data from paper records were abstracted using a standard Microsoft Excel–based data abstraction tool, Ona (Ona, Washington, DC). This included patient demographic characteristics, baseline imaging and pathologic findings, oncologic diagnosis and stage, radiotherapy referral dates, radiotherapy treatment modality and prescription, radiotherapy delays and treatment interruptions, chemotherapy dose and duration, adverse events during the treatment period, and patient status at last follow-up.

### Study Definitions

The radiotherapy referral time was defined as the number of days between intake at BCCOE and the date the patient traveled to Uganda for initiation of radiotherapy. Radiotherapy treatment initiation delays were defined as radiotherapy initiated > 2 weeks from arrival at UCI. Treatment interruption was defined as a delay of 1 day or more beyond the initial treatment plan schedule produced by UCI. Patients were considered lost to follow-up (LTFU) if they first missed a scheduled appointment and then did not return to care after a minimum of three follow-up telephone calls over 6 months.

### Statistical Analysis

Statistical analysis was performed using STATA/SE version 13 (STATA, College Station, TX). For those patients who were not referred for out-of-country radiotherapy, descriptive analysis was conducted to determine demographic information, staging details, and disease profiles. For those patients who received radiotherapy, the same descriptive statistics were calculated; radiotherapy treatment details and early outcomes were also recorded. Median times from diagnosis to event and from completion of radiotherapy to event were calculated for all patients who received radiotherapy. Two approaches were used for these calculations: first, when events included death from any cause, relapsed disease, and LTFU; and second, when events included death from any cause and relapsed disease (LTFU was not considered an event, but was right censored). In both cases, the end time point was either the date of event, if applicable, or the date of data extraction.

### Ethics Approval

The Rwanda National Health Research Committee, the Rwandan National Ethics Committee, the Partners Health Care Institutional Review Board based at Brigham and Women’s Hospital in Boston, MA, and the PIH/Inshuti Mu Buzima Research Committee approved this study.

## RESULTS

### Demographic Characteristics

With OpenMRS, 637 patients were identified as meeting the initial inclusion criteria, and 57 of those patients were subsequently excluded. Reasons for exclusion included the following: 34 patients were excluded because they did not have a documented clinical and/or pathologic diagnosis of cervical cancer, head and neck cancer, or rectal cancer on manual record review; 22 were excluded because their paper medical records could not be located; and one patient was found to have a duplicate record.

Among the final cohort of 580 patients, 497 (86%) were female, and the median age was 52 years (interquartile range [IQR], 44-61.5 years). Nearly all patients (97%) were from Rwanda, 152 (26%) of whom came from the Northern Province where BCCOE is located. The majority of patients, 511 (88%), had Rwandan public medical insurance, known as Mutuelle de Sante. Four hundred twenty-six patients (73%) presented with an ECOG status ≤ 2 (Table 1).

**Table 1:**
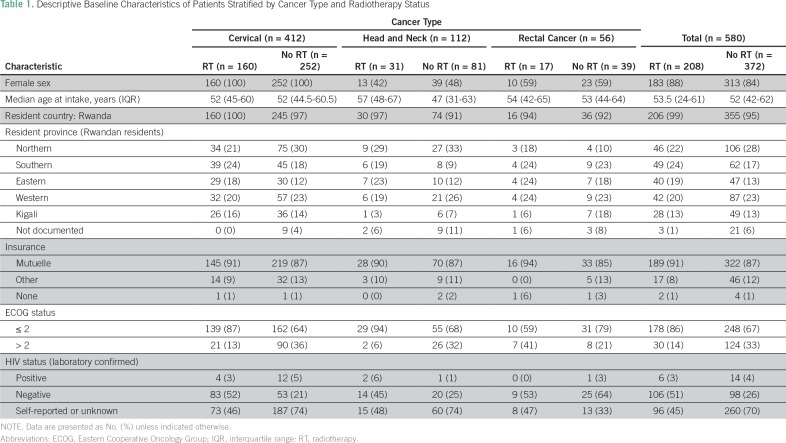
Descriptive Baseline Characteristics of Patients Stratified by Cancer Type and Radiotherapy Status

### Diagnosis and Staging

Regarding diagnosis, 412 (71%) had cervical cancer, 112 (19%) had head and neck cancer, and 56 (10%) had rectal cancer (Table 1). Among patients with cervical cancer, 13 (3%) had stage I, 167 (41%) had stage II, 158 (38%) had stage III, 36 (9%) had stage IV, and 9% did not have a documented stage. Among those with stage III, 57 (36%) had stage IIIa, 84 (53%) had stage IIIb, and 17 (11%) were not specified as type a or b (Table 2). Among patients with head and neck cancer, one (1%) had early-stage disease, 22 (20%) had locally advanced disease, 22 (20%) had metastatic disease, five (5%) had recurrent disease, and 62 (55%) were undocumented. Four patients with rectal cancer (7%) had early-stage disease, 34 (61%) had locally advanced disease, 11 (20%) had metastatic disease, one (2%) had recurrent disease, and six (11%) were undocumented (Table 2).

**Table 2:**
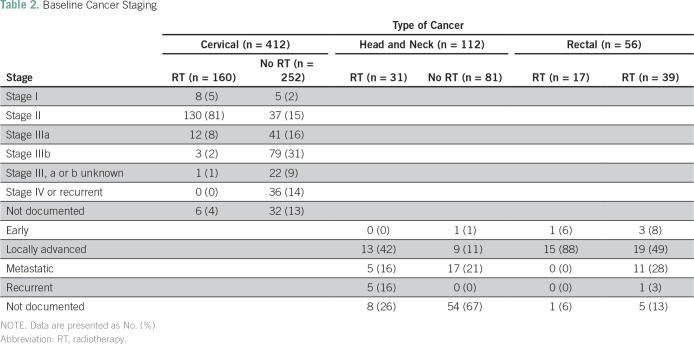
Baseline Cancer Staging

### Radiotherapy Treatment and Follow-Up

Of the 580 patients in the study, 208 (36%) were referred for radiotherapy treatment in Uganda. Of those referred, 160 (77%) had cervical cancer, 31 (15%) had head and neck cancer, and 17 (8%) had rectal cancer. Of the total number of patients with cervical cancer who were seen, 160 (39%) were referred for radiotherapy. Of all patients with head and neck cancer, 31 (28%) were referred, and of all patients with rectal cancer, 17 (30%) were referred (Table 1). Among the referred patients, 172 (83%) had a documented diagnostic biopsy. Staging radiology studies performed before travel to UCI were documented as having been completed for 151 of the radiotherapy patients (73%).

The median referral time was 47 days (IQR, 26-74 days). Once in Uganda, 68 (43%) of the radiotherapy patients were restaged on the basis of UCI’s initial evaluation, inclusive of radiographic studies. The median duration of treatment at UCI was 39 days (IQR, 37-43 days). Nearly all patients (195 [94%]) received concurrent chemotherapy. One hundred thirty patients with cervical cancer (81%) and 16 patients with head and neck cancer (52%) received concurrent cisplatin chemotherapy, and 12 patients with rectal cancer (71%) received fluorouracil and leucovorin chemotherapy. Sixteen percent of patients experienced a treatment delay after arrival, and 27% experienced a treatment interruption (Table 3). Reasons included adverse events and radiation machine maintenance.

**Table 3:**
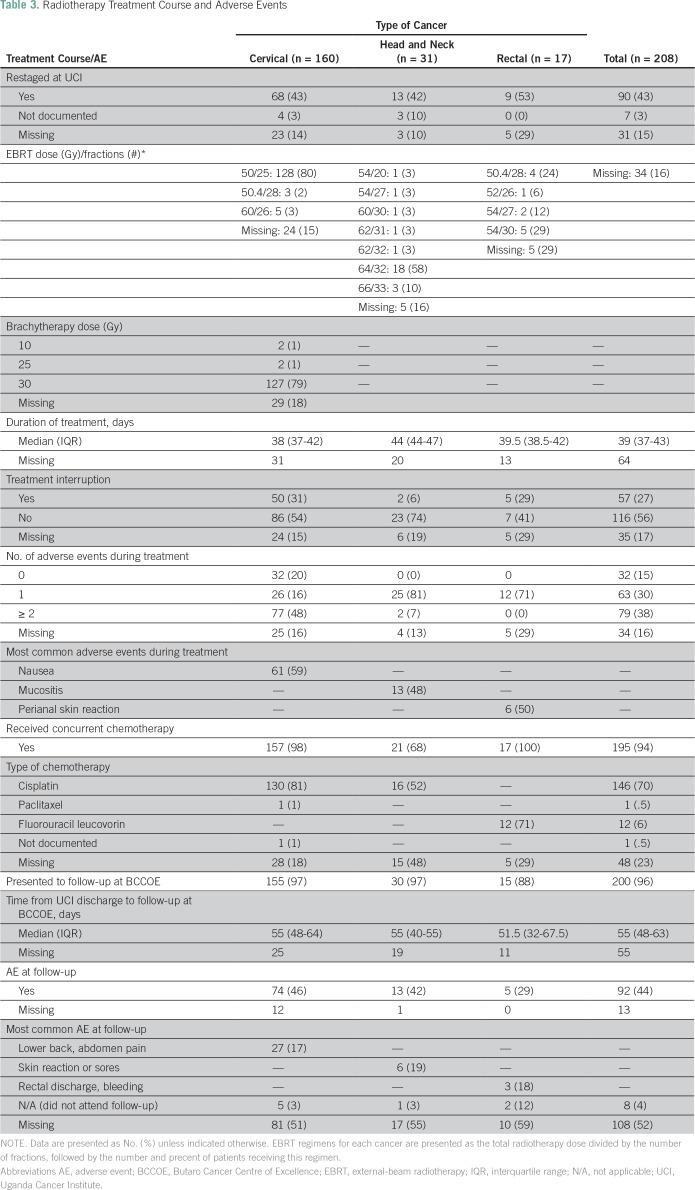
Radiotherapy Treatment Course and Adverse Events

All external beam radiotherapy (EBRT) was delivered with a Cobalt-60 teletherapy unit. EBRT planning was 2-D. Dose and fractionation regimens are provided in Table 3. Brachytherapy for cervical cancer was delivered with a CS-137 source at medium dose rate with tandem and ovoids and prescribed to point A at 0.5 cm from surface. Doses are provided in Table 3. Cisplatin 40 mg/m^2^ was delivered weekly for most patients with cervical and head and neck cancers. For one patient with cervical cancer who could not tolerate cisplatin, paclitaxel 130 mg/m^2^ every three weeks was used. For select patients with advanced head and neck cancer and good performance status (ECOG < or = 1), cisplatin 70 mg/m^2^ every three weeks was used. For patients with rectal cancer, 5-Fu 400 mg/m^2^ and leucovorin 20 mg/m^2^ was given for the first four days and the last four days of radiotherapy. 

### Outcomes

Of those who underwent chemoradiotherapy, 142 (68%) had at least one adverse event documented during treatment. The most common adverse events documented were nausea in patients with cervical cancer (61 patients [59%]); mucositis in patients with head and neck cancer (13 patients [48%]); and perianal skin reaction among patients with rectal cancer (six patients [50%]; Table 3). After completion of radiotherapy treatment, 200 patients (96%) presented for post-treatment follow-up at BCCOE after a median of 55 days (IQR, 48-63 days). Among these patients, 92 (44%) reported at least one adverse event (Table 3).

At the time of data collection, 101 radiotherapy patients (49%) were alive and had completed treatment with no evidence of recurrence, 11 (5%) were alive and continuing their cancer treatment, and 12 (6%) were alive and had completed treatment with evidence of recurrence. Furthermore, 26 (13%) were referred to palliative care for pain and symptom management at their district hospital, 30 (14%) were LTFU, and 19 (9%) were deceased. Specifically, 94 patients with cervical cancer (59%), six patients with head and neck cancer (19%), and one patient with rectal cancer (6%) were alive and had completed treatment with no evidence of recurrence; eight patients with cervical cancer (5%), four patients with head and neck cancer (13%), and seven patients with rectal cancer (41%) were deceased (Table 4).

**Table 4:**
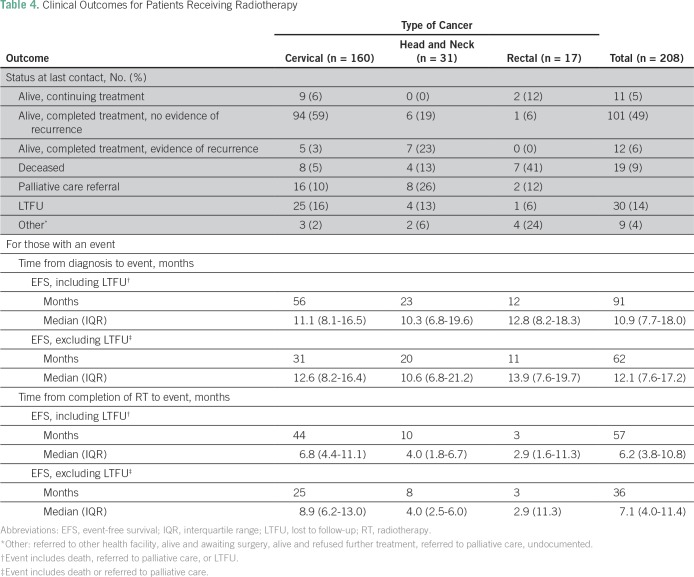
Clinical Outcomes for Patients Receiving Radiotherapy

For all radiotherapy patients, the median time from diagnosis to event (either death, last contact, LTFU, or date of data collection) was 12.2 months (IQR, 8.1-18.6 months); the median time from completion of radiotherapy treatment was 7.9 months (IQR, 4.6-11.5 months; Table 4).

## DISCUSSION

This study demonstrates the feasibility of a rural cancer facility in SSA to successfully implement a protocol-driven, out-of-country radiotherapy referral program. More than one half of the patients who received radiotherapy were alive with no evidence of recurrence at a median follow-up of 12 months, including 59% of all patients with cervical cancer. Although there are few robust data on treatment outcomes in cervical cancer in SSA, this compares favorably to a recent series of patients with cervical cancer in Kenya treated with radiotherapy, which demonstrated an overall survival of approximately 50% at 15 months.^13^ We do note a greater proportion of deceased patients with rectal cancer in comparison with patients with cervical and head and neck cancer. This may be because of the greater proportion of patients with advanced-stage disease, the small sample size, or the requirement for technically advanced surgical interventions for definitive therapy, which is not the case in either cervical or head and neck cancers. Despite only a quarter of patients residing in the Northern Province, retention to care was excellent; 96% of the patients sent for radiotherapy presented at the first follow-up appointment, with a median time of approximately 2 months.

Nevertheless, the results of this study also reveal many lessons learned in providing comprehensive cancer care in resource-limited settings. First, the study reinforced the inherent challenges of providing care to patient populations who initially present at advanced stages of disease. The standard of care for curative treatment of patients who present with late-stage disease is often not available.^14-16^ Identifying and implementing the means for early detection and early stage presentation is critical. BCCOE is piloting a community-based early detection program for breast cancer, with preliminary signs of programmatic success.^17^

Second, many patients lacked proper documentation of pathologic diagnosis or radiographic staging. In part, these results may have been caused by the retrospective nature of this study and the reliance on paper record review. Clinical documents may have been lost after the clinical encounter, or clinicians may have incompletely documented the clinical history and decision-making process. Furthermore, as mentioned previously, the initial protocol did not require all cervical cancer cases to have a pathologic diagnosis.

A third lesson learned is the challenge of treatment delays. There is strong evidence that suggests that the time lapse between diagnosis and the initiation of treatment may significantly affect the outcomes of patients with cancer.^18-20^ In our study, there was an overall median time of 69 days (IQR, 56-109.5 days) between diagnosis and radiotherapy initiation. Delays are in part caused by referrals (CT scan, endoscopy, surgery, and so forth) to Kigali, because they are not available at BCCOE. Furthermore, these delays may explain the upstaging performed at UCI during their initial clinical evaluation.

Concerning the safety of radiotherapy and chemotherapy, there were no deaths attributable to treatment, and the most common adverse events, including nausea, mucositis, and perianal skin reactions, were expected. Regarding concurrent chemotherapy specifically, only two thirds of patients with head and neck cancer were able to receive concurrent cisplatin. As in other settings, patients with head and neck cancer often present with a poor performance status or inadequate renal function, which precludes concomitant chemotherapy. Intensified radiotherapy regimens to compensate for lack of cisplatin were not performed routinely, and cetuximab was not available.

A primary limitation of this study was the lack of a control group. Given the resources available, we analyzed the outcomes of patients sent for radiotherapy but did not analyze patients at BCCOE or other facilities who were unable to undergo radiotherapy. Future studies should include a natural comparison group. Furthermore, the care delivery experience and outcomes at BCCOE may not have comprehensive external validity with respect to other resource-limited settings given the unique factors inherent to the cancer program. These factors include the rural location at a single district hospital and the significant support provided to the Rwandan MOH by PIH and DFBWCC. However, components of the delivery model can provide valuable insights into potential proof-of-concept projects in other settings.

Since the time of data collection, patients from BCCOE have been temporarily no longer able to receive radiotherapy in Uganda because of the inoperability of their cobalt external-beam radiotherapy machine. Patients are now transferred to Nairobi, Kenya, and travel by air. This creates an increase in per-patient cost and, unfortunately, further limits the number of patients able to receive life-saving treatment. The estimated annual cost of the radiotherapy program in fiscal year 2014 was approximately $237,000 USD.^21^ Understandably, many cancer centers in LMICs will not have this resource capacity. This onerous cost has required BCCOE team members and partners to make difficult choices in establishing patient selection criteria, which ultimately determines which patients will be sent for treatment.

Although the ultimate goal is to establish local radiotherapy services, the severe lack of radiotherapy access in SSA demands innovative solutions for those patients in desperate need of curative treatment now. The BCCOE radiotherapy experience illustrates a feasible, short-term solution for those facilities capable of sending patients outside the country. Many lives, especially those with early-stage disease, can be saved now with bridging care until in-country radiotherapy services become available. Fortunately, UCI resumed services in December 2017, and the Rwanda Military Hospital is in the process of establishing new radiotherapy facilities.^22-24^

The BCCOE experience illustrates that a lack of in-country radiotherapy services does not have to be a death sentence. With careful implementation planning, patients with cancer who require life-saving radiotherapy treatment can access care through international referral in a routine, standardized fashion. Other resource-limited settings may be able to apply these lessons to their own diverse settings, so that patients with cancer may have the opportunity for curative treatment despite significant access challenges.
